# Effectiveness of VSV vectored SARS-CoV-2 spike when administered through intranasal, intramuscular or a combination of both

**DOI:** 10.1038/s41598-023-48397-7

**Published:** 2023-12-04

**Authors:** Saina Beitari, Gerard Agbayani, Melissa Hewitt, Diana Duque, Jegarubee Bavananthasivam, Jagdeep K. Sandhu, Bassel Akache, Ita Hadžisejdić, Anh Tran

**Affiliations:** 1 Infectious Diseases, Human Health Therapeutics Research Centre, National Research Council Canada, Ottawa, ON Canada; 2 Immunomodulation, Human Health Therapeutics Research Centre, National Research Council Canada, Ottawa, ON Canada; 3 Preclinical Imaging, Human Health Therapeutics Research Centre, National Research Council Canada, Ottawa, ON Canada; 4 Clinical Department of Pathology and Cytology Clinical Hospital Center Rijeka, University of Rijeka, Rijeka, Croatia

**Keywords:** SARS-CoV-2, Live attenuated vaccines

## Abstract

A critical feature of the VSV vector platform is the ability to pseudotype the virus with different glycoproteins from other viruses, thus altering cellular tropism of the recombinant virus. The route of administration is critical in triggering local and systemic immune response and protection. Most of the vaccine platforms used at the forefront are administered by intramuscular injection. However, it is not known at what level ACE2 is expressed on the surface of skeletal muscle cells, which will have a significant impact on the efficiency of a VSV-SARS-CoV-2 spike vaccine to mount a protective immune response when administered intramuscularly. In this study, we investigate the immunogenicity and efficacy of a prime-boost immunization regimen administered intranasally (IN), intramuscularly (IM), or combinations of the two. We determined that the prime-boost combinations of IM followed by IN immunization (IM + IN) or IN followed by IN immunization (IN + IN) exhibited strong spike-specific IgG, IgA and T cell response in vaccinated K18 knock-in mice. Hamsters vaccinated with two doses of VSV expressing SARS-CoV-2 spike, both delivered by IN or IM + IN, showed strong protection against SARS-CoV-2 variants of concern Alpha and Delta. This protection was also observed in aged hamsters. Our study underscores the highly crucial role immunization routes have with the VSV vector platform to elicit a strong and protective immune response.

## Introduction

SARS-CoV-2, a positive sense, single-stranded RNA virus and the causative agent of COVID-19 has undergone mutations which led to the emergence of variants of concerns (VOCs)^1^. Many different traditional and innovative vaccine strategies and platforms have been utilized for global vaccination campaigns such as protein subunits, inactivated virus, RNA based vaccines, and viral vectors^2^. Majority of vaccines encode the spike antigen of SARS-CoV-2. Spike is a multimeric glycoprotein complex on the surface of the SARS-CoV-2 which mediates the cell attachment by binding to the angiotensin converting enzume-2 (ACE2) receptor. Spike protein is the main target of the protective humoral immune response and virus neutralization; hence, the majority of COVID-19 vaccine developments are focused primarily on spike protein to induce a strong humoral immune response^3^. The emergence of VOCs caused different waves of infection and have contributed to immune escape in the human population with higher transmissibility and fitness. VOC’s exhibit distinct infection patterns including different ACE2 receptor usage patterns, different transmissibility levels and immune evasion^4^. As an example, Alpha has been suggested as most pathogenic compared to Beta, Gamma, and Ancestral strains^5^. Additionally, Delta has been characterized as more transmissible compared to Alpha variant^6^. The more recent Omicron sublineages BA.5 and XBB1.5 are suggested to show greatest immune escape potential compared to their predecessors^1^. While approved mRNA COVID-19 vaccines provide highly effective protection from severe symptoms and reduced risk of transmission, mRNA vaccine-induced immunity is relatively short-lived and variant-specific^7^. The continued evolution of SARS-CoV-2 with potential to acquire mutations conferring greater transmissibility and immune evasion called for an urgent push to develop more effective vaccines that provide longer, efficient protection and also effectively block transmission of emerging variants.

Vesicular stomatitis virus (VSV) is a single-stranded negative-sense RNA virus which belongs to the family of *Rhabdoviridae.* The VSV genome encodes for five major proteins including Nucleoprotein (N), Phosphoprotein (P), Matrix protein (M), Glycoprotein (G), and Polymerase (L)^8^. FDA and EMA approved ERVEBO®, a replication competent recombinant VSV vaccine for Zaire Ebolavirus (EBOV), demonstrates the potential of this vector as a vaccine platform^9^. Furthermore, recombinant VSV has been used as a platform to develop vaccine against other pathogens such as HIV-1 and MERS-CoV^10,11^. The advantage with VSV is the lack of prior immunogenicity in human. VSV is a rare zoonotic infection in human; hence, the prevalence of the naturally acquired VSV antibodies is low in the population and mostly restricted geographically^12^. Notably, deletion of VSV glycoprotein (VSV-G), the major viral antigen targeted by neutralizing antibodies and reported major neurovirulence factor, greatly attenuates the virus and reduces reactogenicity^13,14^. Immunity is not induced against other VSV proteins, permitting multiple administrations with little impact on subsequent protection^15,16^.

A critical feature of the VSV vector platform is the ability to pseudotype the virus with different glycoproteins from other viruses, thus altering cellular tropism of the recombinant virus. The altered cellular tropism should be taken into consideration when determining optimal administration routes of VSV-based vaccines. The route of vaccine administration is critical in triggering a local and systemic immune response and protection^17^. Most of the vaccine platforms used at the forefront are administered by intramuscular (IM) injection. The SARS-CoV-2 VSV-vectored vaccine candidate V590 failed to illicit protective levels of humoral response during Phase I clinical trial may in part potentially be due to the route of administration^18^. A single dose of V590 was administered intramuscularly. ACE2 expression was reported by Li et al. to be low in skeletal muscles but higher in other organs such as heart and kidney^19^. Additionally, Disser et al. demonstrated that only human smooth muscle cells and pericytes express ACE2^20^. The authors demonstrated using single-cell RNA sequencing of human data sets that human skeletal muscle cells, including satellite cells and myofibers, express only TMPRSS2^20^. The potential that minimal expression of ACE2, the cellular receptor for SARS-CoV-2 spike, is found on the surface of skeletal muscle cells^19,21^ may greatly reduce the efficiency of a VSV-SARS-CoV-2 spike vaccine such as V590 when administered intramuscularly.

In this study, we evaluated the immunogenicity and efficacy of replication-competent VSV vector SARS-CoV-2 spike vaccine as single-dose or two-dose regimens administered through 4 different routes and combinations of routes in mice and hamsters: IM, intranasal (IN), IM followed by IN, or IN followed by IM. Intranasal vaccination has been shown to effectively induce secretory IgA response, which is important for conferring mucosal immunity and has the potential to prevent infection and/or transmission^22^. We showed that a single dose administration resulted in very poor antibody response compared to a two-dose vaccination, regardless of vaccination route. Moreover, a two-dose intranasal immunization resulted in the highest IgA response, and both two doses of IN or IM + IN result in the highest IgG detected in BAL samples. Also, IN + IN and IM + IN vaccinated animals showed earlier weight recovery than other tested administration routes and vaccination dose when challenged with SARS-CoV-2 variants of concern Alpha and Delta.

## Methods

### Cells and viruses

HEK293T (Dharmacon, Horizon Discovery, Cambridge, UK), Vero (CCL-81, ATCC), and Vero-TMPRSS2 were maintained in incubators at 34 °C or 37 °C and 5% CO_2_ (depending on the experiment)_._ Cells were maintained in growth media (DMEM supplemented with 1X non-essential amino acids, 1 mM sodium pyruvate, and 10% fetal bovine serum (FBS) was used to maintain the cells). Vero-TMPRSS2 was generated by transduction with lentiviruses generated from vector pLEX307-TMPRSS2-G418 (Addgene plasmid # 158459) and maintained in growth media with G418 (Cat # 15710064, Gibco).

Vesicular stomatitis virus Indiana strain (VSV-WT) was obtained from ATCC (VR-1238). The virus aliquot was amplified on Vero CCL81 cells with infection media (DMEM supplemented with 1X non-essential amino acid, 1 mM sodium pyruvate, and 0.1% bovine serum albumin, 100 Units/mL penicillin, and 100 ug/mL streptomycin). VSV-WT stock was produced by infecting Vero cells at MOI = 0.01. Supernatant was collected at 3 days post infection (dpi), passed through a 0.22 µm filter, and tittered by plaque assay. SARS-CoV-2 variants of concern (VOC) used in hamster challenge study were obtained from BEI Resources: Alpha (hCoV-19/England/204820464/2020, NR-54971) and Delta (B.1.617.2/hCoV-19/USA/MD-HP05647/2021, NR-55672). Each SARS-CoV-2 strain was initially amplified on Vero E6 and titered on Vero cells. Sanger sequencing of S gene confirmed genetic identity to original isolate. All in vivo studies used Delta at passage 3 and Alpha at passage 4.

### VSV-S2 virus generation, rescue, and amplification

Human codon optimized SARS-CoV-2 Spike (Ancestral 2019-nCoV/USA-WA1/2020, GenBank MN985325.1) with 21 deletion in the C-terminus (S2-Δ21) was synthesized by GeneArt (Thermofisher). S2-∆21 was inserted into the VSV-ΔGFP plasmid expression vector (Cat # EH1003, Kerafast Inc). Recombinant VSV-S2 (rVSV-S2) was packaged in HEK293T cells as briefly described: 5 × 10^6^ HEK293T cells were transduced with lentiviral particles expressing T7 polymerase for 24 h, followed by transfection with 10 µg VSV-S2 expression vector and 5 μg of helper expression plasmids VSV-N, VSV-P, VSV-L, and VSV-G (Addgene 64087, 64088, 64085, 8454, respectively). Transfection was done using DharmaFECT kb DNA transfection regents (Cat# T-2006-05, Horizon Discovery). The transfected cells were incubated at 34 °C and supernatant was harvest at 72 h post-transfection and passed through 0.22 μm filter. Rescued virus was amplified in Vero-TMPRSS2 cells with helper plasmid expressing S2-Δ21 for one passage, followed by 5 subsequent rounds of amplification in Vero-TMPRSS2 in absence of helper S2-Δ21 expressing plasmid at MOI 0.1. Passaged 6 rVSV-S2 stocks were titered by plaque assay on Vero-TMPRSS2 cells and used for all subsequent in vitro and in vivo analysis.

### Western blot

Incorporation of SARS-CoV-2 spike into rVSV-S2 was confirmed by western plot. VSV-S2 and VSV-WT viruses were harvested from passage 5 of viral amplification on Vero-TMPRSS2 cells. Cells were infected with VSV-S2 or VSV-WT at an MOI = 0.1 in a 100 mm tissue culture dish. 48 h post-infection VSV-S2 or VSV-WT virus particles were pelleted by ultracentrifugation for 2 h, 35,000 RPM, at 4 °C. Viral pellets were lysed in 4X Laemmli buffer. Lysates were run on 10% SDS–polyacrylamide gel and transferred onto polyvinylidene difluoride (PVDF) membrane. Membrane blots were blocked in 5% skim milk/TBST (Tris-buffered saline with 0.1% Tween 20 (Cat# 194841, MP BioMedicals)) for 1 h at room temperature, followed by detection with primary antibodies mouse anti-S1 antibody (Cat# MAB105403, R&D systems), mouse anti VSV-N (Cat # MABF2348, Sigma Aldrich), or mouse anti-VSV-G (Cat #MABF2321, Sigma Aldrich). Blots were then treated with secondary rabbit anti-mouse IgG-HRP (Cat#610-4302, Rockland) and developed with enhanced chemiluminescence (ECL) reagents (catalog number NEL105001EA; PerkinElmer). Blot was imaged on Bio-Rad ChemiDoc XRS + imaging system with parameters “Chemiluminescence, High Resolution.” Exposure times are indicated in figure legends.

### Animal study

All animals used in this study were maintained at the small animal facility of the National Research Council Canada (NRC) in accordance with the guidelines of the Canadian Council on Animal Care. All procedures performed on animals in this study were in accordance with regulations and guidelines reviewed and approved in animal use protocol 2020.06 by the NRC Human Health Therapeutics Animal Care Committee. All experiments were conducted in accordance with relevant guidelines and regulations, including the ARRIVE guidelines.

To determine humoral and cell-mediated immune response to VSV-S2, a total of 70 female hACE2-KI mice between 6–7 weeks old (Jackson Laboratory, strain 035,000) were randomly divided into 8 vaccination groups (n = 10 for VSV-S2, n = 5 for VSV-WT control). On day 0 (D0), animals lightly anaesthetized with isoflurane then vaccinated either IM or IN (as indicated) with VSV-S2 or VSV-WT at 1 × 10^6^ PFU per animal. Groups receiving 2 vaccination doses were given boosters on D21. Animals were euthanized D35 and tissues collected to assess immunogenicity. Serum was collected on D-2 (prebleed), D20 and D35 post-vaccination.

Animal challenge study was conducted with a total of 85 male Golden Syrian hamsters (81–90 g, 6–7 weeks old; Charles River Laboratory). Hamsters were randomly divided into 14 vaccination groups (n = 6 for VSV-S2, n = 5 for VSV-WT control). For the aged cohort, a total of 14 hamsters were used (12–18 months old), randomly divided into 2 groups (n = 7). On D0, hamsters lightly anaesthetized with isoflurane then vaccinated with VSV-S2 or VSV-WT via IM or IN route at 1 × 10^6^ PFU per animal. Animals receiving 2 vaccination doses, received the booster on D28 by IM or IN. Serum was collected on D-2 (prebleed), D27, and D55. Immunized hamsters were divided into two groups on D56. Animals were anesthetized by injection of Ketamine/Xylazine (90 kg/mg/8 kg/mg) and intranasally challenged with either SARS-Cov-2 Alpha (hCoV-19/England/204820464/2020, B.1.1.7) or Delta (hCoV-19/USA/MD-HP05647/2021, B.1.617.2) at 8.5 × 10^4^ PFU per animal. Weight and clinical symptoms were monitored daily. Animals were humanely euthanized by CO_2_ five days-post infection (dpi) and tissues immediately collected for subsequent analysis.

### Anti-spike IgG and IgA ELISA

Total Spike-specific IgG titers in the serum and bronchoalveolar lavage (BAL) were quantified by ELISA. Briefly, 96-well high binding ELISA plates were coated overnight at room temperature with 100 µL of 0.3 µg/mL resistin-trimerized Spike (SmT1) protein diluted in PBS. After washing plates with PBS/0.05% Tween20 (PBS-T; Sigma-Aldrich, St. Louis, MO, USA), wells were blocked for 1 h at 37 °C with 200 µL 10% fetal bovine serum (Thermo Fisher Scientific) in PBS. Serum samples were serially diluted 3.162-fold and added to the plates to allow for binding of antibodies to the protein for 1 h at 37 °C. Bound IgG was incubated with goat anti-mouse IgG-HRP (1:4000, Southern Biotech, Birmingham, AL, USA) for 1 h at 37 °C prior to washing plates 5 times with PBS-T and adding 100 µL o-phenylenediamine dihydrochloride substrate (Sigma-Aldrich). After incubating for 30-min at room temperature while protected from light, 50 µL stop solution (4 N H_2_SO_4_) was added to each well. Bound IgG Abs were detected at 450 nm using the FilterMax F5 microplate reader (Molecular Devices, San Jose, USA). Anti-Spike IgG titers in the serum were defined as the dilution that resulted in an absorbance value (OD450) of 0.2 and were calculated using XLfit software (ID Business Solutions, Guildford, UK). Samples that did not reach the target OD were assigned the value of the lowest tested dilution (i.e. 10) for analysis purposes. No detectable titers were measured in serum samples from negative control animals.

Total Spike-specific IgA titers in the serum and BAL were quantified by ELISA, as described previously^23^. Briefly, 96-well high binding ELISA plates were coated overnight at 4 °C with 100 µL of 0.8 µg/mL SmT1 protein diluted in PBS. Plates were washed five times with PBS-T, and then blocked for 2 h at 37 °C with 200 µL 3% Difco™ skim milk (BD) in PBS. Serum samples were serially diluted 3.162-fold and added to the plates to allow for binding of antibodies to the protein for 1 h at 37 °C. Bound IgA was incubated with goat anti-mouse IgA-HRP (1:10,000, Abcam, Cambridge, UK) for 45 min. at 37 °C prior to washing plates 5 times with PBS-T and adding 100 μL KPL SureBlue™ Tetramethylbenzidine (TMB) microwell peroxidase substrate (one-component) (Mandel Scientific Company Inc., Guelph, ON, Canada). The reaction was stopped by adding 100 μL KPL TMB Stop solution (Mandel Scientific Company Inc.) after a 10 min. incubation at room temperature while protected from light. Bound IgA Abs were detected at 450 nm, and IgA antibody titers were determined as above for IgG.

### IFN-γ ELISpot

Total Spike-specific cell numbers were enumerated by ELISpot using a mouse IFN-γ kit (Mabtech Inc., Cincinnati, OH, USA). Splenocytes were stimulated with the PepMix™ SARS-CoV-2 Spike Glycoprotein peptide library (315 peptides; 15mers overlapping by 11 amino acids) (PM-WCPV-S-2; JPT Peptide Technologies GmbH, Berlin, Germany), which was split into 2 subpools (158 + 157 peptides). Each subpool was separately used to stimulate 4 × 10^5^ cells in duplicate at a final concentration of 2 µg/mL per peptide. Cells incubated without any stimulants were used to measure background responses. Spot-forming cells (SFCs) were counted using an automated ELISpot plate reader (Cellular Technology LTD, Beachwood, OH, USA). For each animal, values obtained with media alone (background) were subtracted from those obtained with each of the Spike peptide pools, and then combined to yield an overall number of antigen-specific IFN-γ^+^ SFCs/10^6^ splenocytes per animal.

### RBD-specific IgG ELISA

RBD-specific IgG titers in collected serums were quantified using ELISA. Briefly, Nunc MaxiSorp flat-bottom 96 well plates were coated with SARS-CoV-2 RBD- His recombinant protein (40,595-V80H, Sino Biological, China) and incubated overnight at 4 °C. Following incubation, plates were washed with PBS containing 0.1% Tween-20 (PBS-T) and blocked with 3% Bovine Serum Albumin (IgG-Free). Fivefold serially diluted hamster serums were transferred to the plate and incubated for 1 h at 37 °C. Plates were then washed with PBS-T, and Peroxidase AffiniPure Goat Anti-Syrian Hamster IgG (H + L) (Cat # 107-035-142, Jackson Immuno Research, West Grove, USA) was added to each well and incubated at 37 °C for 1 h. After the last wash with PBS-T, 100 µL of TMB substrate (Cat# 7004P6, Cell Signaling Technology, MA, USA) was added to each well and incubated at room temperature for 2 min before 100 ul of TMB Stop solution (Cat# 7002P6, Cell Signaling Technology, MA, USA) was added. Absorbance was measured at 450 nm. Inhibitory dilution 50 (ID50) was calculated using non-linear regression analysis.

### Plaque assay

Infectious viral load was quantified by plaque assay on homogenized lung and nasal turbinate tissues as previously described^24^. In brief, spin-clarified supernatants of tissue homogenates were serially diluted 1:10 in infection media (DMEM supplemented 1X non-essential amino acid, 1 mM sodium pyruvate, and 0.1% bovine serum albumin). Dilutions were adsorbed on Vero cells for 1 h at 37 °C/5% CO_2._ After adsorption, inoculum was removed and 1 ml of infection media with 0.6% ultrapure, low-melting point agarose was overlaid over the cell monolayer and incubated for 72 h. To visualize plaques, cells were fixed with 10% formaldehyde and stained with crystal violet. Plaques forming units (PFU) was determined per gram tissue.

### Plaque reduction neutralization assay

The PRNT assay was performed in the NRC’s CL3 facility as previously described^5^. Serum samples were inactivated at 56 °C for 30 min and stored on ice. The inactivated serum was serially diluted 1-in-2 and incubated with equal volume of 100 PFU of SARS-CoV-2 at 37 °C for 1 h, followed by infection of Vero E6-ACE2-TMPRSS2 cells (NIBSC, Great Britain, cat# 101003). Adsorption of virus were carried out for 1 h at 37 °C. After adsorption, inoculum was removed and cells were overlaid with media as described above. The assay was incubated at 37 °C/5% CO_2_ for 72 h (or 96 h for BA.5 virus). Cells were fixed with 10% formaldehyde after incubation and stained with crystal violet. No serum, virus-only back-titer control was included along with naïve animal serum. PRNT50 is defined as the highest dilution of serum that results in 50% reduction of plaque-forming units. The 1-in-2 dilution of diluted serum to 100 PFU virus was included in the final calculation.

### Real time quantitative-PCR

Viral RNA from hamster respiratory tissues including lung and nasal turbinate were extracted using Quick-viral RNA kit according to the manufactures (Cat #R1035, Zymo Research, Irvine, USA). Luna Universal One-step RT-qPCR kit was used to quantify viral genomic RNA (Cat #, E3005S, New England Biolabs, MA, USA) with primer/probe sets for the SARS-CoV-2 E gene^25^ (Forward: 5′ACAGGTACGTTAATAGTTAATAGCGT Reverse: 5′ ATATTGCAGCAGTACGCACACA Probe: ACACTAGCCATCCTTACTGCGCTTCG 5′Fam 3′QSY-1). Known concentrations of viral RNA copies were used to generate standards. 5 μL of extracted RNA were run in duplicate on Applied Biosystems StepOne Real time PCR (ThermoFisher Scientific, MA, USA) and results analyzed with StepOne Software.

### Histology

The left lobes of infected hamster lungs were isolated and fixed in 10% formalin for 1 week at room temperature. Fixed tissues were transferred into 70% ethanol, then processed and embedded in paraffin wax. The paraffin block was cut into 4 µm sections and placed on Superfrost Plus slides (Fisher Scientific). Sections were dried, and duplicate sections were subjected to hematoxylin and Eosin (H&E) or immunohistochemical (IHC) staining. H&E staining was done on a fully automated Leica ST5010-CV5030 system; SARS-CoV-2 nucleocapsid was detected with mouse anti-SARS-CoV-2 nucleocapsid monoclonal antibody (1:5000, R&D System MAB10474) on the Bond-Max III fully automated staining system (Leica Biosystems, Wetzlar) with a modified F protocol and Bond Polymer Refine Detection. Following deparaffinization and rehydration, sections were pre-treated with the Epitope Retrieval Solution 1 (ER1, Citrate buffer, pH 5.0) at 98 °C for 20 min. After washes, non-specific endogenous peroxidases were quenched using peroxidase block for 5 min. Sections were washed again and then incubated for 15 min at room temperature with primary antibodies. Sections were washed again and incubated with polymer refine for 8 min at room temperature and developed with 3, 3’-diaminobenzidine (DAB) chromogen for 10 min. After final wash sections were counterstained with hematoxylin for 5 min, dehydrated, cleared, and mounted. Negative controls included omission of primary antibody and incubation with secondary antibody alone as well as lung tissue from naïve animals.

Stained slides were scanned at 20 × magnification using a Zeiss Axio Scan.Z1 digital slide scanner capable of brightfield imaging, and analysed on QuPath 0.3.2 (https://qupath.github.io)^26^. Briefly, images were then set to brightfield (H-DAB), and immunopositive (stained with hematoxylin and DAB) and negative cells (stained with hematoxylin only) were selected. Immunopositive cells were determined with in-software function *analyze_cell detection_positive cell detection_run*. Values were then exported into an excel file and the data therein was used for graphing results. Parameters for optimal cell detection and analysis such as threshold value etc. were determined beforehand for each antibody stain and those values were kept constant for all sections.

### Statistical analysis

Data were analyzed using GraphPad Prism 9 (GraphPad Software, Boston, MA, USA). Statistical significance of the difference among groups was determined through one-way analysis of variance (ANOVA) or two- way ANOVA (as indicated in the figure legend) with Tukey’s multiple comparison test (comparison across all groups). ELISA and ELISpot data were log-transformed prior to statistical analysis. Significant differences were considered with p < 0.05. Statistical significance was indicated as follows: *p < 0.05, **p < 0.01, ***p < 0.001 and ****p < 0.0001.

## Results

### Generation and rescue of replication competent, recombinant VSV expressing SARS-CoV-2 spike

**Figure 1:**
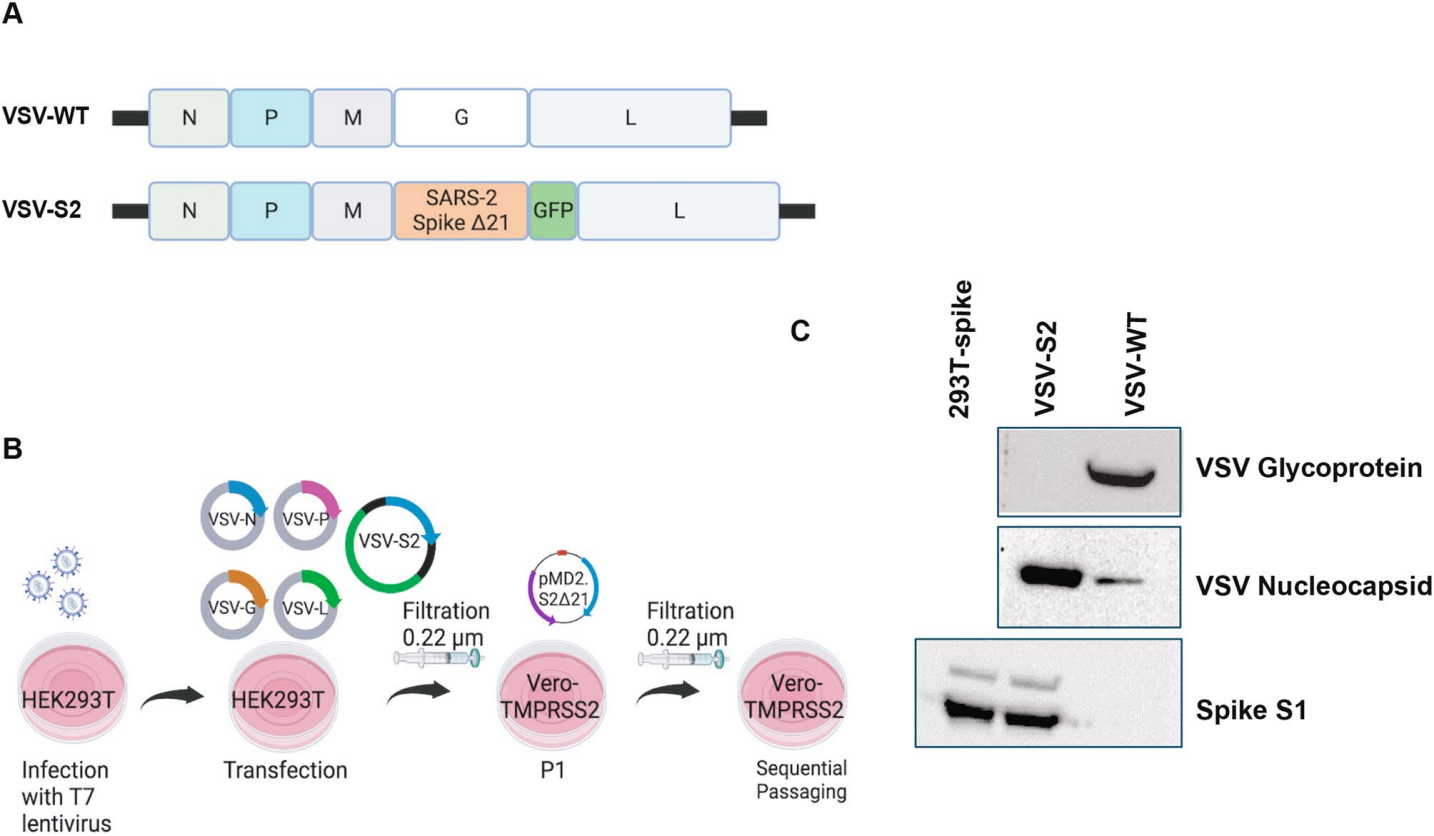
VSV-S2 recombinant virus rescue amplification. (**A**) Illustrative genome for VSV- WT and VSV-S2. N: Nucleocapsid, P: Phosphoprotein, M: Matrix, G: Glycoprotein, L: Polymerase. To generate VSV-S2, Glycoprotein G is replaced with SARS-2 Spike Δ21. (**B**) Schematic illustration of VSV-S2 viral rescue and propagation. (**C**) Western blot for detection of viral proteins VSV-G, VSV-N, and SARS-CoV-2 spike S1. All lanes were run on the same gel and transferred to the same blot. The blot was subsequently cut into three sections immediately after protein transfer to membrane. Each section was then blocked with skim milk and detected with mouse anti-VSV nucleocapsid, mouse anti-VSV-G antibodies, or mouse anti-SARS-CoV-2 spike S1, respectively. Blots were imaged at an exposure of 10 s. Blots were cropped and resized for clarity and presentation. No other alteration of image was made. Original, uncropped and full size image of blots are shown in Supplementary Fig. S1. Plasmid expression of SARS-CoV-2 spike in 293 T was included as a positive control. Supernatants containing VSV-S2 or VSV-WT were harvested from Vero-TMPRSS2 cells (MOI = 0.1) 48 h post infection. Prior loading on SDS-PAGE gel, supernatants were filtered and centrifuged.

The VSV vector vaccine expressing SARS-CoV-2 spike (VSV-S2) was generated by replacing VSV-G in the vector with a codon-optimized SARS-CoV-2 spike harbouring a 21 amino acids (Δ21) C-terminal deletion (Fig. 1A). Studies showed that the C-terminal truncation is necessary for efficient incorporation of the spike into virus vectors^27,28^. After the primary rescue of the virus on HEK293T cells transduced with T7 polymerase lentivirus; Vero cells expressing TMPRSS2 were used to propagate the virus by sequential passaging (Fig. 1B). First round of VSV-S2 amplification was conducted on Vero-TMPRSS2 cells expressing Spike Δ21 as a complement and helper plasmid. Subsequent five passages were carried out on Vero-TMPRSS2 in absence of exogenous Spike Δ21 expression. Incorporation of SARS-CoV-2 spike in VSV particles and expression in infected Vero-TMPRSS2 cells was confirmed by western blot (Fig. 1C, Supplementary Figs. S1–S3). Lysate from 293 T cells expressing SARS-CoV-2 spike was included as a positive control. As expected, unlike VSV wildtype (WT), VSV-S2 does not express VSV glycoprotein (G).

### A two-dose vaccination induced stronger SARS-CoV-2 spike-specific IgG response in hACE2-KI mice than a single dose administration

**Figure 2:**
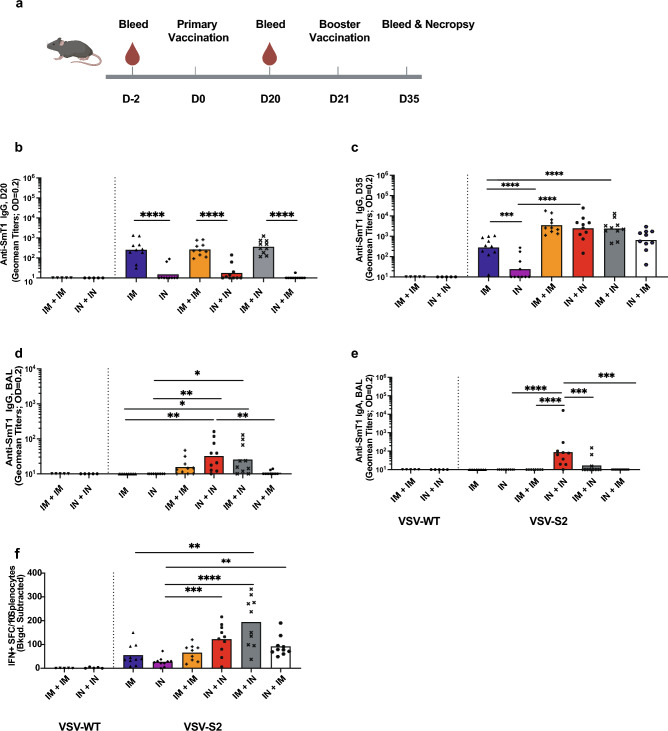
VSV-S2 induced both humoral and cell-mediated immunity in hACE2-KI mice using different combination of administration route. (**a**) Schematic figure of immunogenicity study plan. (**b**) Serum spike-specific IgG titer of all cohorts at day 20 post primary vaccination. (**c**) Booster dose was administered in appropriate animal cohorts as indicated and serum spike-specific IgG titer was determined for all cohorts at day 35 post vaccination. (**d**) BAL spike-specific IgG titer on day 35 post-vaccination. (**e**) BAL spike-specific IgA titer determined on day 35 post-vaccination. (**f**) T cell response was determined by IFN-γ ELISpot on Day 35 post-vaccination. n = 5 for VSV-WT, n = 10 for VSV-S2, each spot represents one animal sample. Antibody titers are presented as geometric means. All other data are presented as means. Statistical significance of the difference among groups was determined through one-way analysis of variance (ANOVA) followed by Tukey’s multiple comparison test (comparison across all groups. For all analyses, differences were considered to be significant with p < 0.05. Statistical significance was indicated as follows: *p < 0.05, **p < 0.01, ***p < 0.001 and ****p < 0.0001.

To assess the immunogenicity of VSV-S2 vaccine and to evaluate the mucosal immunity induced by VSV-S2, we compared the immunogenicity of single-dose IM or IN, or 2-dose heterologous route of vaccination in hACE2- K1 mice. Mice were randomly divided into groups of 10 mice, where 2 groups were given either a single dose by IM or IN with VSV-S2, 2 groups were vaccinated with VSV-WT control (n = 5), and the remaining groups were administered with 2-doses given 21 days apart. Different combinations of routes of administrations in the prime-boosted groups were investigated as indicated in Fig. 2a.

Spike specific IgG titer in serum on day 20 (D20) and day 35 (D35) post vaccination was detected using ELISA to evaluate systemic immune response (Fig. 2b,c). A single dose intramuscular delivery resulted in significantly higher spike-specific IgG titers in mice on D20 compared to IN vaccinated groups (Fig. 2b). However, mice that received a booster dose showed elevated levels of spike-specific IgG compared to the single-dose vaccinated groups (Fig. 2c). The increase in IgG titer was similar for all boosted cohorts independent of whether the first dose was delivered intramuscularly or intranasally. However, animals that were first vaccinated intranasally and boosted intramuscularly showed a slightly lower IgG titer, albeit the difference is not significant.

We further assessed spike-specific IgG titers in BAL samples collect at D35 post-vaccination (Fig. 2d). Single-dose immunization of VSV-S2 as well as 2-doses delivered by IM + IM and IN + IM elicited very poor IgG levels in BAL. Highest IgG titers were determined in animals prime-boosted by routes IN + IN and IM + IN (Fig. 2d). Mice vaccinated with VSV-WT control did not show any detectable anti-SARS-CoV-2 spike IgG (Fig. 2b–d).

### Mucosal IgA was strongly detected in the 2-dose, intranasally vaccinated mice

Given the critical role IgA antibodies play in mucosal immunity, we next evaluated the IgA response in BAL samples collected from vaccinated mice by ELISA on day 35 post vaccination (Fig. 2e). Single-dose immunization with VSV-S2 resulted in undetectable levels of IgA titer. A similar response was observed in animals prime-boosted intramuscularly. Most interesting was the low response observed in animals prime-boosted IM + IN or IN + IM. In contrast, a 2-dose delivery all done intranasally resulted in the strongest IgA response compared to all other groups of vaccinated animals.

### Intramuscular followed by intranasal prime-boost induced greatest cell-mediated response in hACE2-KI mice

Cell-mediated immunity is a critical arm of host immunity against viral infections, and this was assessed by IFN-γ ELISpot conducted on splenic lymphocytes harvested on D35 post vaccination and stimulated for 24 h with peptide pools spanning the spike protein (Fig. 2f). Single-dose of VSV-S2 delivered intranasally resulted in the lowest levels of IFN-γ as compared to the other vaccinated groups including the single dose intramuscularly vaccinated animals. Interestingly, 2-dose delivery of VSV-S2 all done intramuscularly did not show greater IFN-γ production compared to the single-dose IM vaccinated group. We observed that, while not statistically significant, IN + IN, IM + IN and IN + IM vaccination regimens induced a slightly higher IFN-γ response, especially in animals that were vaccinated first intramuscularly and boosted intranasally.

### Two-dose IN or IM + IN administration elicited greatest protection in vaccinated young adult hamsters

**Figures 3:**
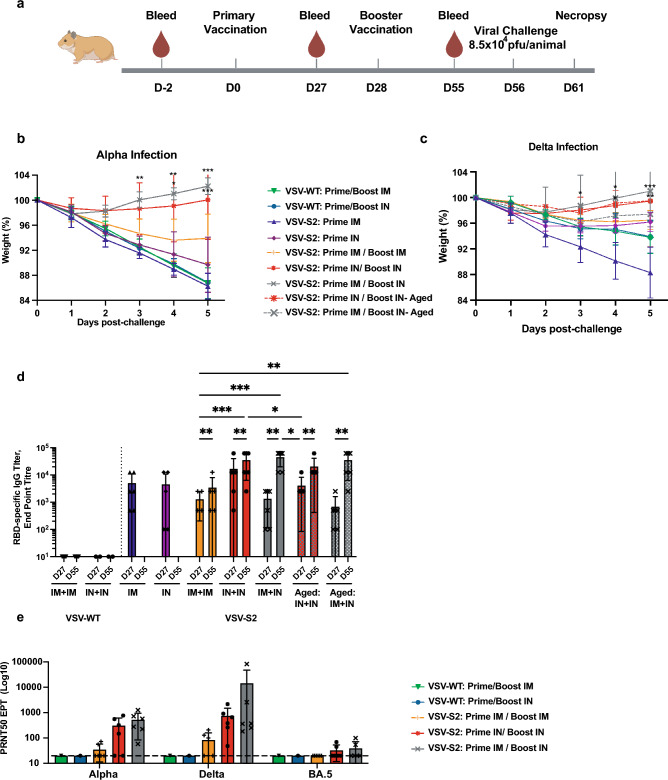
Golden Syrian hamsters vaccinated with VSV-S2 using different combination of administration routes were protect from challenge with Alpha or Delta variant. (**a**) Schematic figure of efficacy study design using Golden Syrian hamsters. (**b**) Body weight was monitored in vaccinated hamsters challenged with Alpha. Day 0 corresponds to the day of challenge with SARS-CoV-2 Alpha. (**c**) Body weight was monitored in vaccinated hamsters with Delta. The Delta study includes the aged cohort (VSV-S2: Prime IN/ Boost IN- Aged, VSV-S2: Prime IM/ Boost IN- Aged). (**d**) Serum RBD-specific IgG titers were determined on day 27 and day 55 post-primary vaccination using ELISA. For body weight analysis, two-way analysis of variants (ANOVA) followed by Tukey’s multiple comparison test was used to determine significant differences. The significant differences indicated on the graph represent the comparisons between VSV-S2 vaccinated cohort and the VSV-WT control group. (**e**) PRNT was conducted using hamster serums vaccinated with VSV-S2 or VSV-WT and collected on day 55 post primary vaccination. The 50% plaque reduction neutralization test (PRNT50) is defined as the highest serum dilution resulting in 50% reduction in plaque formation unit. LOD is 40 (represented with broken horizontal line). For graphing purpose, anything below 40 is set as 20, indicating below LOD and non-neutralizing. For ELISA analysis, ordinary one-way ANOVA followed by Tukey’s multiple comparison test was used to determine significant differences. Data shown is mean ± SD of 6 hamsters per VSV-S2 group and 5 hamsters per VSV-WT groups. For all analyses, differences were considered to be significant with p < 0.05. Statistical significance was indicated as follows: *p < 0.05, **p < 0.01, ***p < 0.001 and ****p < 0.0001.

We wanted to determine the efficacy of VSV-S2, expressing a spike protein from the Ancestral strain, in conferring cross-protection to VOC strains. We approached this two ways: (1) to determine the effectiveness of the vaccine candidate, and (2) to evaluate the effect different combinations of IN and IM vaccination routes have on this vaccine’s efficacy. Golden Syrian hamsters were vaccinated with VSV-S2 or VSV-WT using different immunization routes and combinations of routes as indicated in Fig. 3a. After 56 days post vaccination, the hamsters were intranasally challenged (8.5 × 10^4^ PFU/animal) with Alpha or Delta. In Alpha infected animals, VSV-WT vaccinated hamsters showed a 14% weight loss on day 5 post infection, regardless of the route of vaccination. VSV-S2 when delivered by IM + IN or IN + IN protected animals challenged with Alpha (Fig. 3b). These animals recovered with weight gain as early as day 2 post-infection. Interestingly, IM + IN showed a slightly better weight recovery than IN + IN, though this is not statistically significant. Contrastingly, animals primed and boosted intramuscularly continued to lose up to 7% of their weight by day 5 post infection. Single dose immunization with VSV-S2 did not protect hamsters from infection and resulted an observed weight loss of 11% and 14% in IN and IM groups, respectively (Fig. 3b).

Vaccinated animals challenged with Delta showed similar levels of protection as observed in vaccinated, Alpha challenged cohorts. Again, hamsters vaccinated with VSV-S2 by IM + IN or IN + IN was protected from Delta infection and showed no weight loss as early as day 2 post-infection (Fig. 3c). Single-dose vaccination by IN showed partial protection with less weight loss than IM vaccinated animals, 4% vs 12% body weight, respectively. These results strongly demonstrate that VSV-S2 is most effective when delivered as a 2-dose regimen either by IM + IN or IN + IN.

### Two-dose administration elicited strong protection in vaccinated aged hamsters

Aged cohorts are at greater risk of infection and disease severity due to waning immunity. We evaluated the effectiveness of VSV-S2 to protect aged hamsters (12–18 months old) exposed to Delta. The animals were vaccinated with a 2-dose regimen both delivered intranasally or the initial dose delivered intramuscularly with the second dose given intranasally. Following booster vaccination, the animals were infected with Delta (Fig. 3c). Vaccination with two doses, regardless of administration route, resulted in minimal weight loss in the aged cohort. Notably, IN + IN immunized aged hamsters showed a level of protection on par with those observed in young vaccinated IN + IN animals at D5 post-challenge, where the animals regained an average of 99% of their original weight. Interestingly, an observable difference in the level of protection was noted in young vs aged cohorts vaccinated by IM + IN. While IM + IN still offered some protection in aged animals compared to the control group, we still observed about 2.6% weight loss at D5 post-challenge compared to the young, vaccinated cohort which gained 101% of their initial weight during the same time period (Fig. 3c).

### IM + IN and IN + IN vaccination routes induced strong RBD-specific IgG response in both young and aged animals

We next assessed the effect of different routes of administration and one versus two-dose regimen in eliciting protective levels of RBD-specific IgG in the serum of vaccinated animals as measured by ELISA (Fig. 3d). No significant difference in RBD-specific IgG titers were observed between single dose delivery by IM or IN (Fig. 3d). Booster vaccination via IN route significantly increased the RBD-specific IgG titers in hamsters (Fig. 3d). Both groups IM + IN and IN + IN exhibited stronger RBD-specific IgG titer compared to IM + IM. Aged cohorts vaccinated with VSV-S2 also showed superior RBD specific IgG titers compared to IM + IM group. The control groups did not show any detectable RBD-specific IgG titer.

We next determined whether the IgG titer corresponds to antibody neutralizing activity to Alpha, Delta, and the more recent Omicron sub lineage BA.5. We focused our neutralization assay on sera from animals vaccinated with two doses, which conferred the highest antibody response. While we saw an overall trend of higher PRNT50 titer in the IN + IN and IM + IN, the differences between vaccination routes were not statistically significant (Fig. 3e). However, the ability for antibody neutralization of BA.5 was greatly reduced compared to Alpha and Delta. This was expected given that the vaccine antigen was based on the Ancestral spike protein.

### Complete clearance of infectious virus burden was achieved in the upper respiratory tracts of both young and aged vaccinated animals

**Figure 4:**
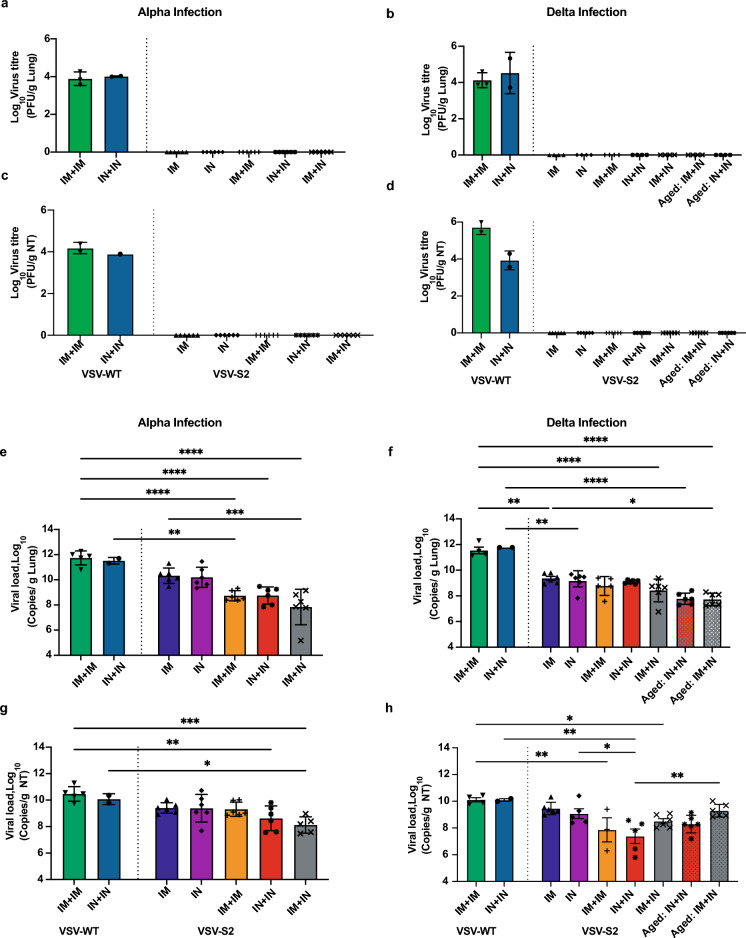
VSV-S2 vaccinated hamsters cleared infectious virus in respiratory tissues. Infectious viral titer in (**a**) lung of vaccinated hamsters challenged with Alpha, (**b**) lung of vaccinated hamsters challenged with Delta, (**c**) nasal turbinate (NT) of vaccinated hamsters challenged with Alpha, and (**d**) NT samples of vaccinated hamsters challenged with Delta. Genomic viral RNA burden was determined by RT-qPCR in lung tissues from hamsters infected with (**e**) Alpha or (**f**) Delta. Genomic viral RNA burden was determined by RT-qPCR in NT from hamsters infected with (**g**) Alpha or (**h**) Delta. Ordinary one-way ANOVA followed by Turkey's multiple comparison test was used to determine significant differences. Data shown is mean ± SD of 6 hamsters per VSV-S2 group and 5 hamsters per VSV-WT groups. For all analyses, differences were considered to be significant with p < 0.05. Statistical significance was indicated as follows: *p < 0.05, **p < 0.01, ***p < 0.001 and ****p < 0.0001.

An important aim for vaccination is to minimize the viral burden in the respiratory tracts. We sought to determine the viral load in lungs and nasal turbinate of vaccinated and infected hamsters by plaque assay. No infectious viruses were detected in the VSV-S2 vaccinated hamsters at day 5 post-challenge, regardless of the route of vaccination or the number of administered doses (Fig. 4a–d). However, both groups of VSV-WT vaccinated hamsters (IM + IM or IN + IN) showed high viral burden in the respiratory tissues (Fig. 4a–d). To further confirm our observation, we next determined the amount of viral RNA in lungs and nasal turbinate of these vaccinated hamsters. In vaccinated animals challenged with Alpha, the 2-dose regimen resulted in significantly lower levels of viral RNA in the lungs compared to the control VSV-WT group, while elevated levels of viral RNA remained in the single-dose vaccinated cohort (Fig. 4e). Notably, the IM + IN vaccinated group was significantly superior in reducing viral RNA levels in the lungs infected with Alpha variant compared to the single-dose IM cohort. In contrast, all vaccinated animals that were challenged with Delta, including the aged cohort, showed significantly lower levels of RNA in the lung compared to the control VSV-WT group, regardless of the route of vaccination or the number of doses (Fig. 4f). Unlike in the Alpha-challenged cohort, we did not observe significant differences in viral RNA burden between IM + IN and the single-dose IM cohorts.

Similarly, reduced amounts of viral RNA were observed the nasal turbinate of Alpha infected animals vaccinated by routes IN + IN and IM + IN but not in the IM + IM or single dose IN or IM cohorts (Fig. 4g). While we also observed reduced viral RNA burden in the nasal turbinate of vaccinated animals challenged with Delta, the greatest reduction was observed in animals given 2-doses, both intranasally (Fig. 4h).

**Figure 5:**
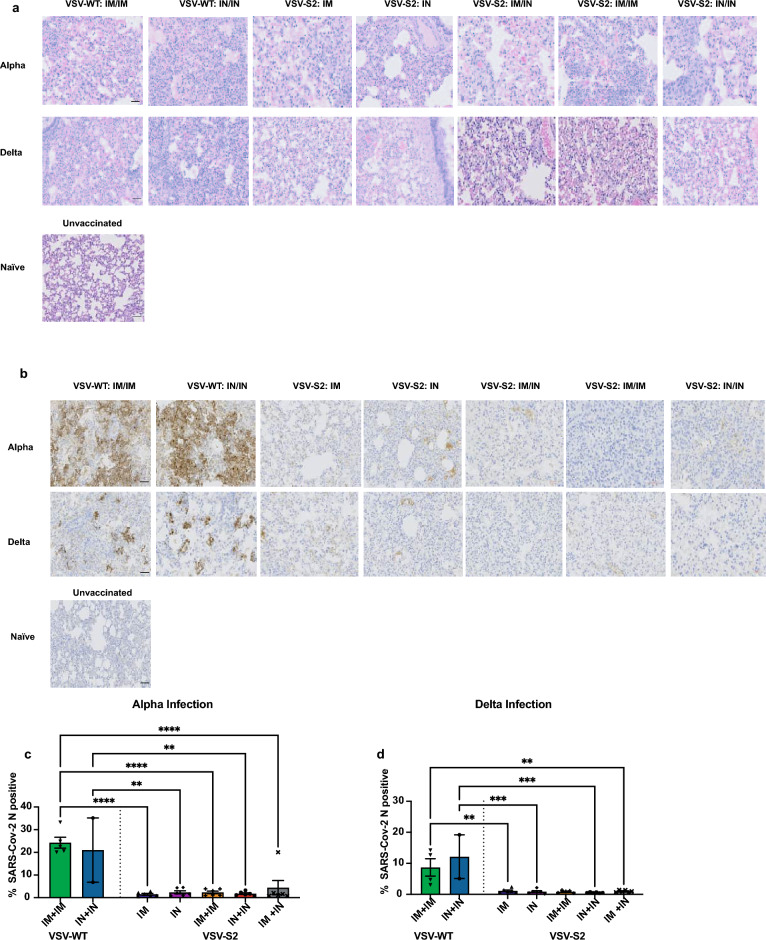
Histopathology and immunohistochemistry of hamster lungs vaccinated with VSV-S2 showed reduced inflammation and viral burden. Hamsters were challenged on day 56 post-vaccination with SARS-CoV-2 Alpha or Delta, and lung tissues were collected at 5 days post-challenge. (**a**) Lung tissue sections were stained by hematoxylin–eosin (H&E). (**b**) Lung tissues were stained with anti-SARS-CoV-2 nucleocapsid (N). (**c**,**d**) Quantitative analysis of nucleocapsid protein staining on IHC images from infected lungs at day 5 post challenge using Qupath software as described in “Materials and methods” section. Data shown is mean ± SD of 6 hamsters per VSV-S2 group and 5 hamsters per VSV-WT groups. *indicates p-value < 0.05; **indicates p < 0.01; ***indicates p-values < 0.001 (bar = 100 μM).

We performed histopathological examination of lung from hamsters vaccinated with VSV-WT and VSV-S2 on 5 days post infection with Alpha or Delta. While we did not observe substantial differences in lung pathology between VSV-WT control and VSV-S2 vaccinated hamsters, or between the different vaccination routes. VSV-WT vaccinated hamsters did show noticeably higher levels of consolidations and cell infiltrations in the airway (Fig. 5a). IN vaccinated hamsters or hamsters boosted intranasally such as IM + IN and IN + IN groups also showed milder lung histopathology.

Immunohistochemistry (IHC) was done on lung samples of vaccinated hamsters with an antibody specific to SARS-CoV-2 nucleocapsid. Lung samples revealed high levels of viral nucleocapsid staining in VSV-WT control animals infected with Alpha or Delta (Fig. 5b,c). However, tissues from vaccinated animals showed low to undetectable nucleocapsid stains, regardless of the routes of administration or the number of doses administered (Fig. 5b,c). Similar to Alpha infected animals, VSV-S2 vaccinated hamsters infected with Delta did not show any significant nucleocapsid staining (Fig. 5b,d). These overall findings strongly support vaccinated animals achieved high level of protective immunity to Alpha or Delta infections in the respiratory tissues.

## Discussion

Since the beginning of the pandemic, different VSV based vaccine vectors expressing spike protein (full length or truncated) have been studied and shown to protect hamsters and mice from SARS-CoV-2 infection by inducing a strong immunity^29–35^. Two of these VSV based vaccine vectors expressing spike protein led to clinical trials. The first one is IIBR-100 which is currently on Phase 2b/3 clinical trials (https://clinicaltrials.gov/ct2/show/NCT04990466). The other one is V590 vaccine which is produced by Merck^36^. The Phase I clinical trial of a single dose of V590 had to be terminated due to insufficient immune responses produced by the participants (https://www.clinicaltrials.gov/ct2/show/NCT04569786). Notably, in this clinical trial, only IM route of vaccination had been explored. Similarly, most of the approved COVID-19 vaccines are intramuscularly administered. Vaccines administered by IM have demonstrated a high degree of efficacy and induce strong humoral and cell mediated immune responses^37–40^. However, studies have shown that IM vaccine delivery results in poor mucosal response such as IgA, which is a critical component in blocking viral transmission^41–43^. Unlike other virus vector platforms, exogenous virus glycoproteins completely replace the VSV-G in the VSV-∆G vector, in which the VSV glycoprotein is deleted. This changes the host cell tropism of the recombinant VSV (rVSV). We suspect that a part of the poor immune response induced by Merck’s V590 in their Phase 1 clinical trial may be due to the route of vaccination. ACE2 was reported to have low levels of expression in skeletal muscles but higher in other organs such as heart and kidney^19^, Disser et al. demonstrated that only human smooth muscle cells and pericytes express ACE2^20^. The authors demonstrated with single-cell RNA sequencing of human datasets that human skeletal muscle cells, including satellite cells and myofibers, express only TMPRSS2^20^. The potential that minimal expression of ACE2, the cellular receptor for SARS-CoV-2 spike, is found on the surface of skeletal muscle cells^19,21^ may greatly reduce the efficiency of a VSV-SARS-CoV-2 spike vaccine such as V590 when administered intramuscularly. A recent study by Taddeo et al.^29^ showed an optimized and enhanced IM route of VSV based viral vector spike vaccination using trans-complemented with the VSV-G glycoprotein. VSV-G is known to have broad tissue tropism. The authors also demonstrated that VSV SARS-CoV-2 construct without the VSV-G helper did not induce protective immunity following intramuscular immunization. Together, this further suggests that the efficacy of the VSV-S2 is dependent on the tissue tropism of the inserted virus glycoprotein, and further emphasized the importance of the administrative route for this platform.

In this study, we examined the affect of a single dose or two dose delivery of a VSV expressing SARS-CoV-2 spike by intranasal, intramuscular, or a combination of both. We observed that a two-dose delivery of VSV-S2 resulted in higher IgG response compared to one dose, with more elevated IgG levels detected in BAL. Interestingly, the IM two-dose group showed equal IgG titer to the other vaccinated groups in hACE2-KI mice. However, we observed a noticeable difference in RBD-specific IgG response in hamsters for the IM + IM cohort. The antibody response induced by VSV-S2 in IM + IM group of hACE2-KI mice showed IgG titers which is similar to spike specific titers induced by IM + IN or IN + IN animals. However, hamsters immunized with 2 doses intramuscularly (IM + IM cohort) showed significantly lower IgG titer compared to other vaccinated groups. This raises the possibility that there may innate differences between the transgenic knock-in mice and hamsters, underscoring caution when using transgenic animals. Notably, IgA response was observed highest in IN + IN immunized animals. Minimal IgA was detected in IM + IN vaccinated animals but none were detected for other cohorts. Evaluation of neutralizing IgG activity in the sera of two-dose vaccinated hamsters showed a muted ability to neutralize Omicron BA.5 for all tested cohorts, regardless of vaccination dose or delivery route. However, the same sera showed strong neutralizing titer to Alpha and Delta. This observation was expected since the VSV-S2 vaccine encodes for the Ancestral spike. While immune escape was reported for Delta^44^, it is more so in recent SARS-CoV-2 variants such as Omicron^44–46^. Neutralization titer was comparable for IN + IN and IM + IN but both were slightly higher than IM + IM.

Single dose or two dose intramuscular delivery showed comparable T cell response, determined by measurement of IFN-γ ELISpot. However, significantly more elevated IFN-γ was detected in IM + IN vaccinated animals compared to single dose delivery by IM or IN, and trended higher when compared to two dose delivery by IN or IM (5 out of 10 mice vaccinated by IM + IN showed more elevated T cell response compared to IN + IN and IN + IM). In contrast, T cell response in IN + IM vaccinated animals showed no difference when compared to IM + IM and IN + IN.

A two-dose immunization, especially delivered IN + IN or IM + IN, facilitated earlier and better recovery, as demonstrated by strong weight gain as early as 2 days post-challenge. IM + IN and IN + IN cohorts showed consistently better weight recovery when challenged with Alpha or Delta. By day 5 post-challenge, most animals in the IN + IN and IM + IM cohorts fully recovered their original weight or more, while weight loss was still observed for animals in the other treatment groups. However, by day 5 post-challenge, all animals, regardless of vaccination dose or delivery route, showed full clearance of infectious virus load in the respiratory tracts. This suggests that one or two doses of VSV-S2, delivered via any of the tested administration routes, provided some degree of protection to vaccinated animals, both old and young.

Interestingly, despite comparable spike-specific IgG titers between young and aged animals, IM + IN vaccinated aged animals did not show the same level of weight recovery as observed in the young cohort by 5 days post-challenge with Delta. In contrast, IN + IN vaccinated animals showed comparable weight recovery in both young and aged animals. Intranasal vaccination has the advantage of also inducing strong mucosal IgA immunity, which may provide better upper respiratory tract protection and reduce the ability of the virus to establish an infection^47^. IM + IN vaccinated young animals did not demonstrate similarly strong IgA responses. Immunosenescence has a pivotal role in impairing immune response to vaccination and rendering older individuals vulnerable to infections^48^. Two studies reported an increase in salivary IgA secretions as well as comparable IgA levels in the guts of senior human individuals versus young adults, while also noting significant decline in IgG titers in these aged individuals^49,50^. While we did not determine IgA titer in the aged animals, we suspect IgA mucosal immunity may play a strong role in protecting the aged cohort from Delta infection. This may explain, in part, the comparable weight recovery between young and aged animals when vaccinated IN + IN but not IM + IN. In contrast, IM + IN vaccinated young animals tended to show a slightly better weight recovery and T cell response than other treatment groups. This may suggest that strong T cell activity in young animals may have a significant role in protection from virus infection. Further studies are required to better understand the individual roles the mucosal and systemic immune responses have in protecting senior and young adult populations.

We have shown here that immunization with VSV expressing SARS-CoV-2 spike demonstrated strong protection when animals were vaccinated with two doses by IN or IM + IN. However, all vaccination routes and a single dose of VSV-S2 still resulted in some degree of protection. Our study highlights a need to better understand how the VSV vector behaves in the presence of different virus glycoproteins and when these are administered through different vaccination routes. While our study was limited to intranasal and intramuscular delivery, other vaccinations routes such as subcutaneous, intradermal, and intravenous should also be investigated. These studies will provide valuable information toward the design of future VSV vector vaccines and immunization regimens. Our report also highlights the need to investigate the impact of different routes of vaccination on aged populations and whether specific delivery routes are more effective in certain age groups.

### Supplementary Information

Supplementary Figures.

## Data Availability

The data presented in this study are available on reasonable request from the corresponding author.
