# Supplementary material for: Effectiveness of VSV vectored SARS-CoV-2 spike when administered through intranasal, intramuscular or a combination of both
Source: Sci Rep. 2023 Dec 4;13:21390. doi: 10.1038/s41598-023-48397-7 (PMC10695950; doi:10.1038/s41598-023-48397-7)
Supplement: Supplementary file 1 — Supplementary Figures. [file 41598_2023_48397_MOESM1_ESM.docx]

**VSV-WT**

**VSV-S2**

**a**

150-

100-

75-

63-

48-


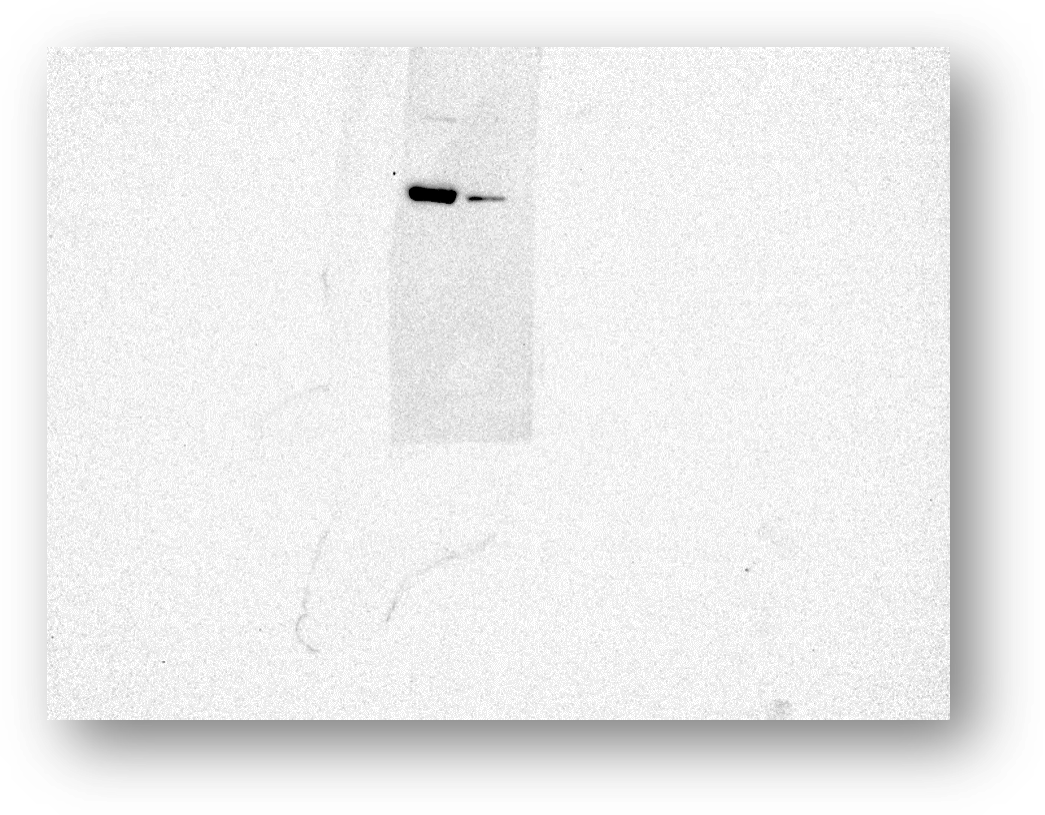


**VSV-WT**

**VSV-S2**

**b**

150-

100-

75-

63-

48-


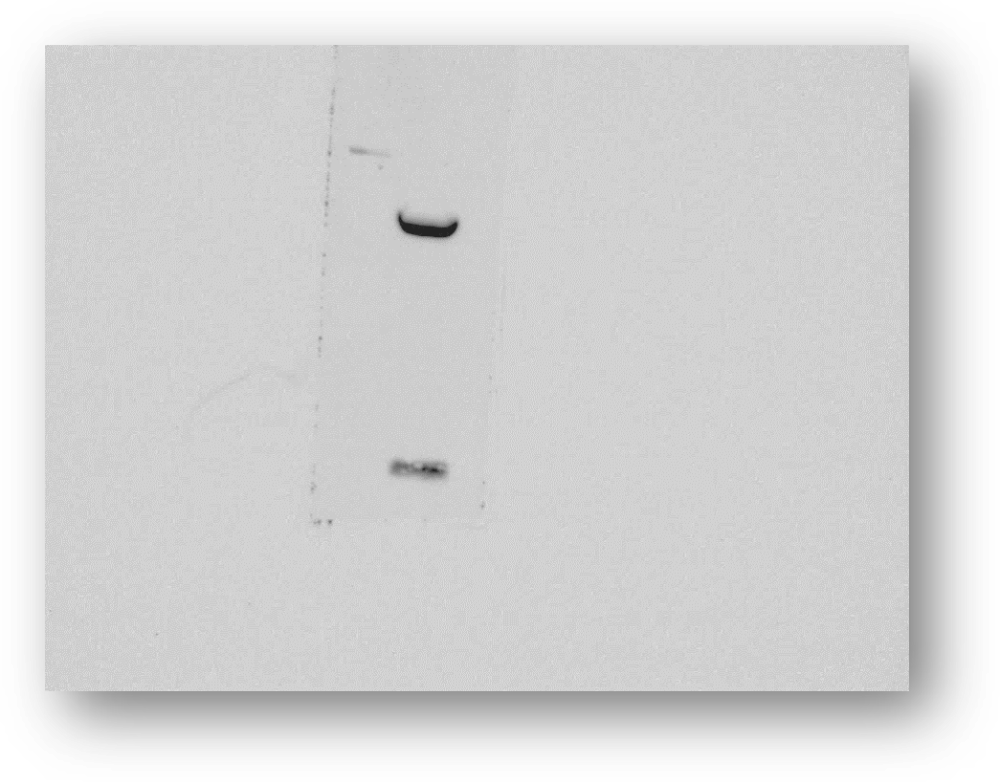


**VSV-S2**

**VSV-WT**

**293T-Spike**

150-

100-

75-

63-

48-


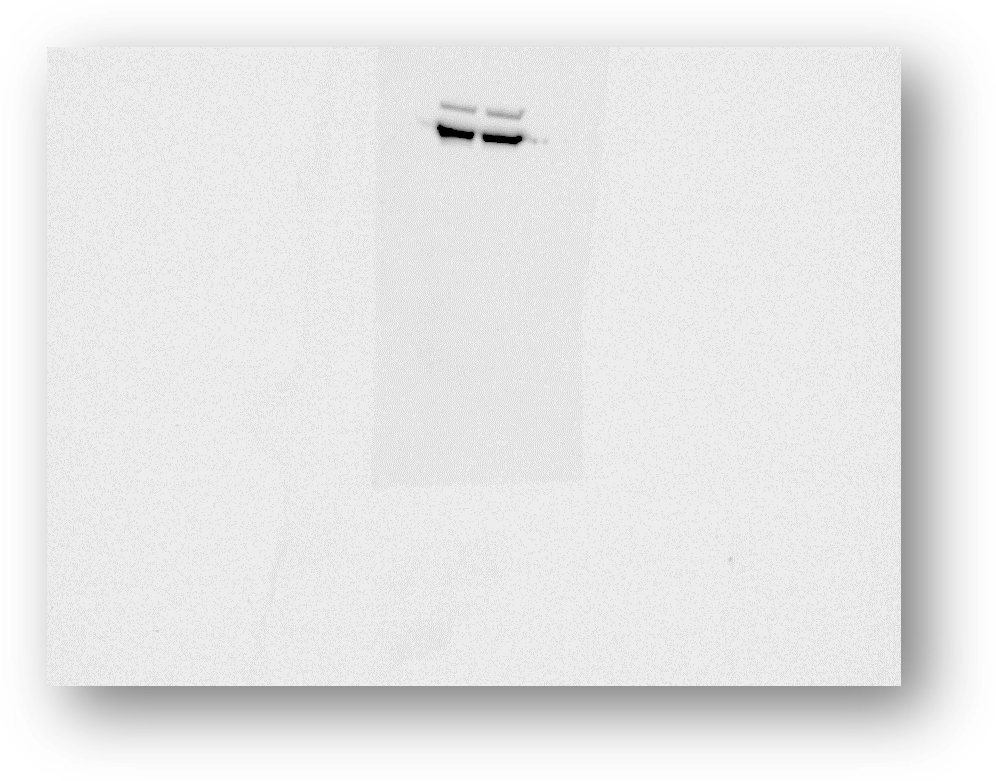


**c**

**Supplementary Figure S1. Unedited, original images of western blots shown in Figure 1c.** All lanes were run on the same gel and transferred to the same blot. The blot was subsequently cut into three sections immediately after protein transfer to membrane. Each section was then blocked with skim milk and detected with a) anti-VSV nucleocapsid, b) anti-VSV-G antibodies, and c) anti-SARS-CoV-2 spike S1. Images shown correspond to Fig. 1c. Western blot for detection of viral proteins VSV-G, VSV-N, and SARS-CoV-2 spike S1. Plasmid expression of SARS-CoV-2 spike in 293T was included as a positive control. Supernatants containing VSV-S2 or VSV-WT were harvested from Vero-TMPRSS2 cells (MOI=0.1) 48 hours post infection. Prior loading on SDS-PAGE gel, supernatants were filtered and centrifuged. Dash outline indicates membrane edges.

**VSV-WT cell lysate**

**VSV-S2 cell lysate**

**VSV-S2 particle**

**VSV-WT particle**

175-

80-

46-

30-

58-


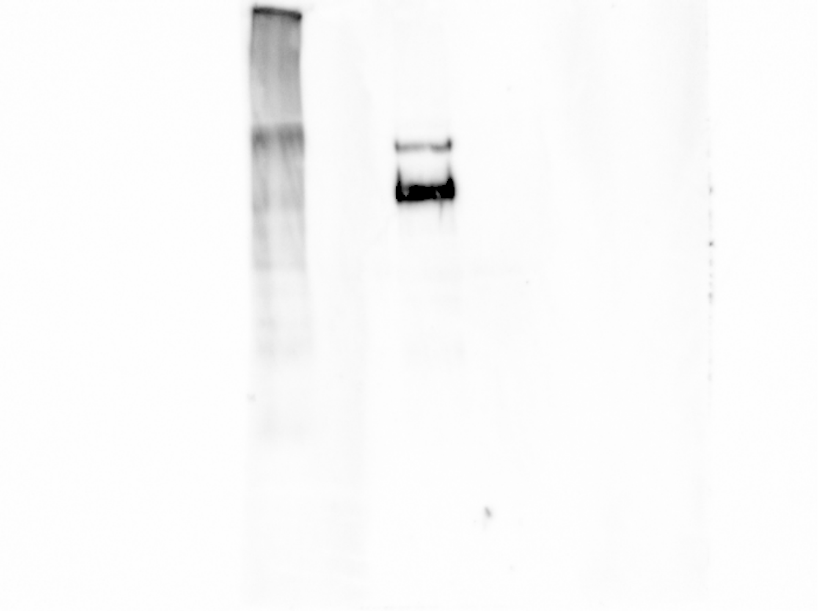


**a**

FL

S1

**VSV-WT cell lysate**

**VSV-S2 cell lysate**

**VSV-S2 particle**

**VSV-WT particle**

175-

80-

46-

30-

58-


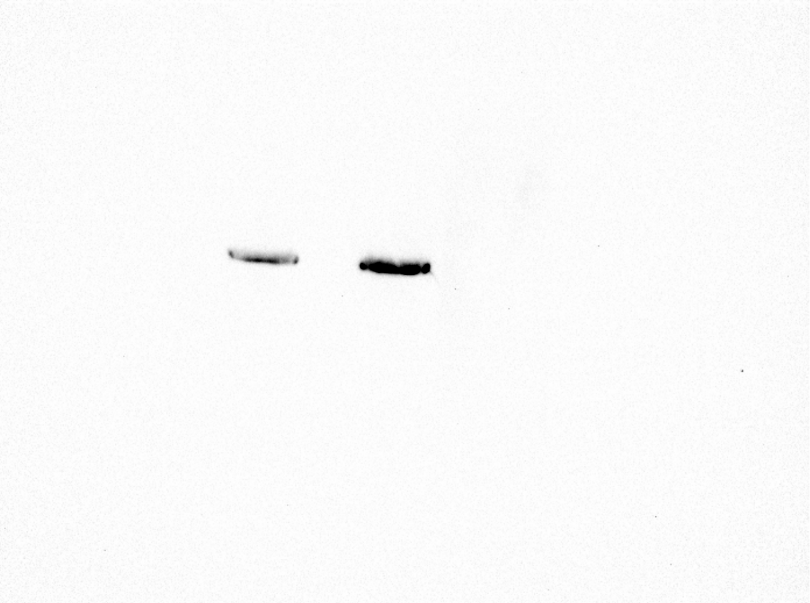


**b**

**VSV-WT cell lysate**

**VSV-S2 cell lysate**

**VSV-S2 particle**

**VSV-WT particle**

175-

80-

46-

30-

58-


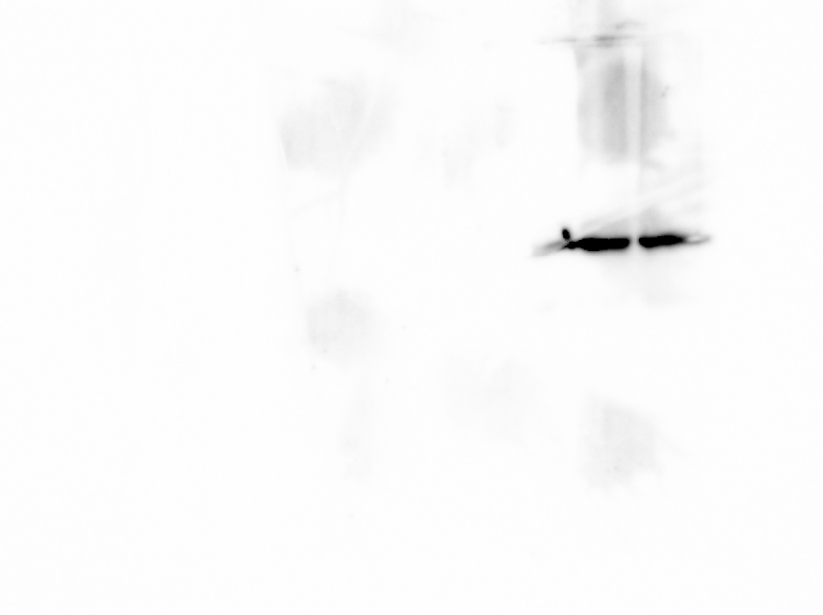


**c**

**Supplementary Figure S2. SARS-CoV-2 spike expression in Vero E6 cells infected with VSV-S2.** Vero E6 cells were infected with VSV-S2 or VSV-WT at MOI 1 and cell lysates harvested 48 h post-infection into 200 μl RIPA Lysis and Extraction Buffer (ThermoFisher cat# 89900). Samples were heated at 100^o^C for 10 min and 50 μl was loaded per lane. VSV-S2 or VSV-WT virus stocks, pelleted from pre-filtered supernatant as described in the Methods section was loaded at 20 μl per well. Samples were run in triplicate as three separate gels and transferred to PVDF blot membranes. Membrane blots were probed with a) mouse anti-SARS-CoV-2 S1 spike antibody (Cat# MAB105403, R&D systems), b) mouse anti-VSV-G (Cat #MABF2321, Sigma Aldrich), and c) mouse anti-β-actin (GenScript cat #A00702). Blots were then treated with secondary rabbit anti-mouse IgG-HRP (Cat# 610-4302, Rockland) and developed with enhanced chemiluminescence (ECL) reagents (catalog number NEL105001EA; PerkinElmer). Blots were imaged on Bio-Rad ChemiDoc XRS+ imaging system. Shown are unedited, original blot images. Resized to fit within body text. Same unedited, original blot images with multiple exposure times are presented in Supplementary Figure S4. FL designates full length SARS-CoV-2 spike. S1 designates the S1-containing domain of spike.


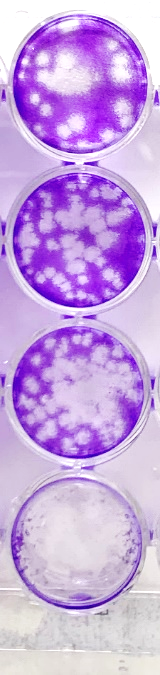

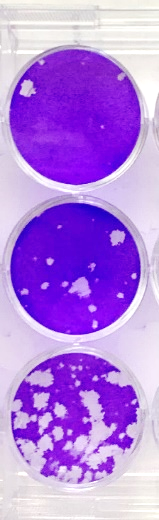


VSV-S2

VSV-WT

10^-4^

10^-5^

10^-6^

10^-5^

10^-6^

10^-7^

**Supplementary Figure S3. VSV-S2 titer by plaque assay.** Virus stocks were serially diluted 1:10 in infection media (DMEM supplemented 1X non-essential amino acid, 1 mM sodium pyruvate, and 0.1% bovine serum albumin). Dilutions were adsorbed on Vero cells for 1 hour at 37оC/5% CO2. After adsorption, inoculum was removed and 1 ml of infection media with 0.6% ultrapure, low-melting point agarose was overlaid over the cell monolayer and incubated for 72 h. Cells were fixed with 10% formaldehyde and stained with crystal violet. Plaques forming units (PFU) per mL was determined.

**a**


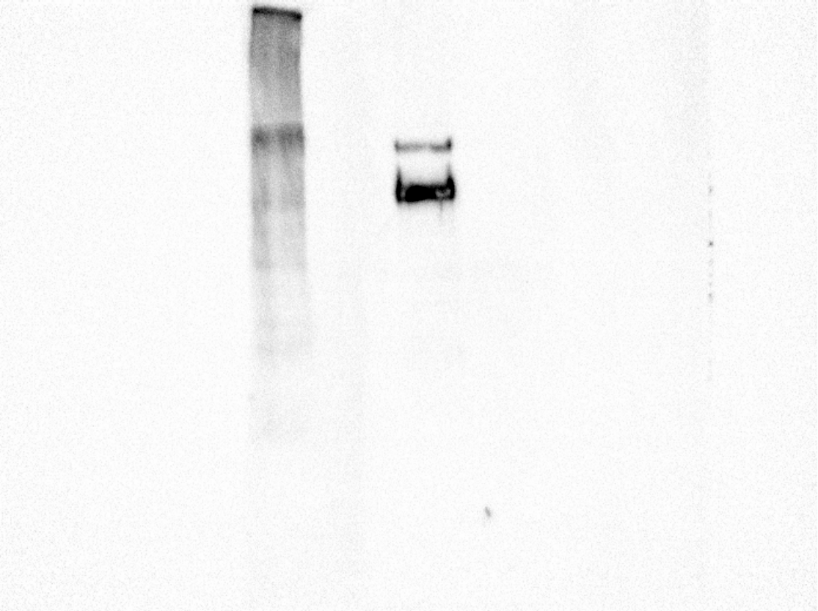


Exposure: 0.1 sec


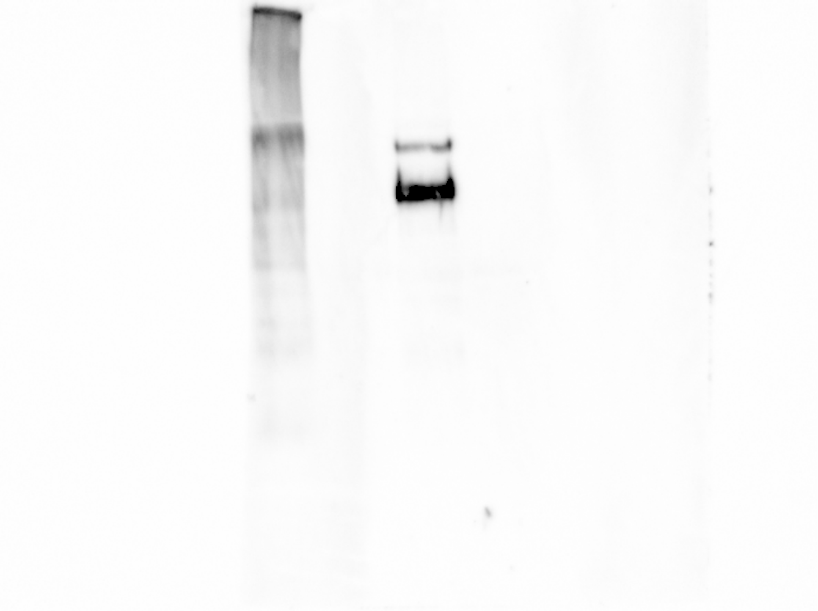


Exposure: 1 sec


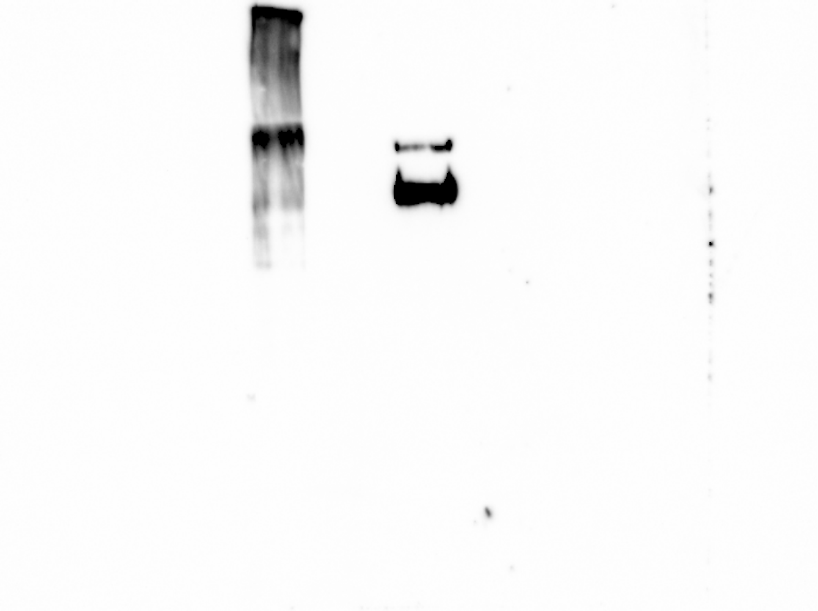


Exposure: 5 sec

**b**


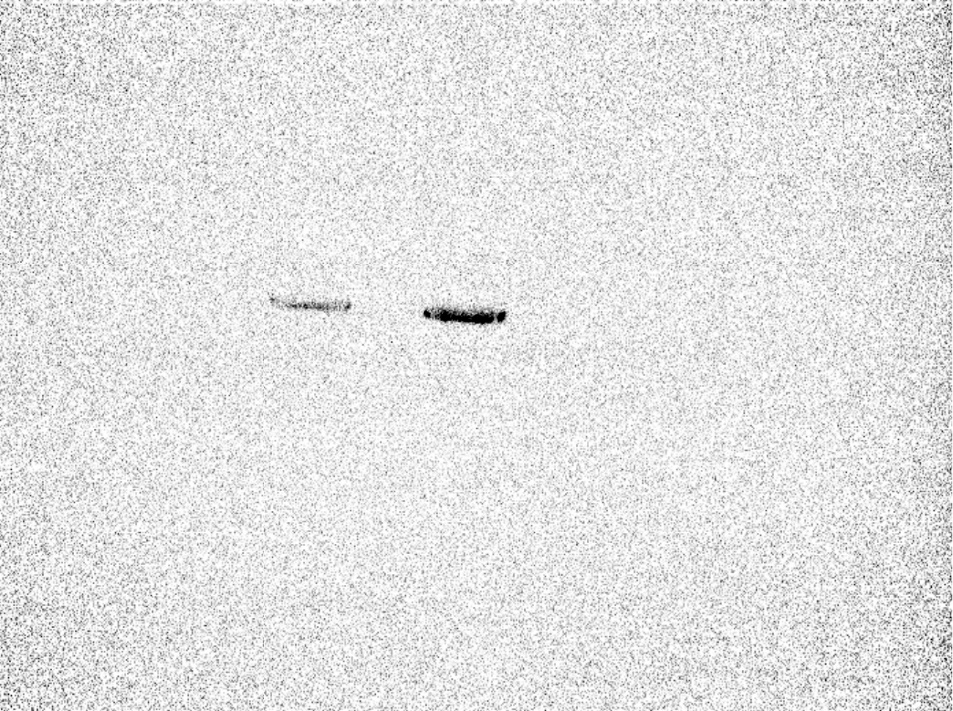


Exposure: 1 sec


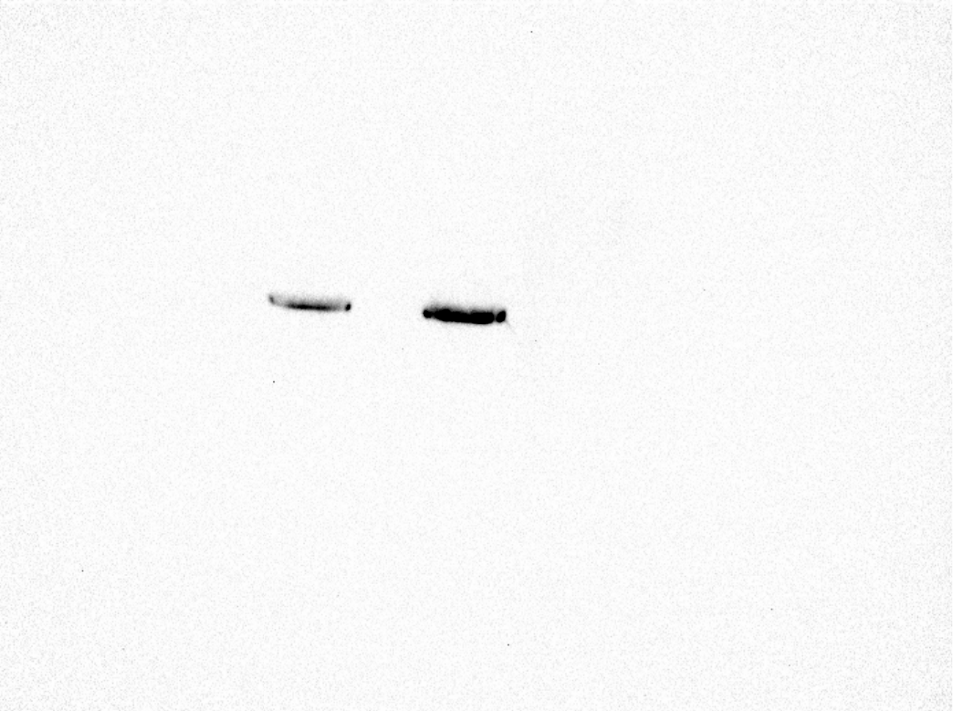


Exposure: 10 sec


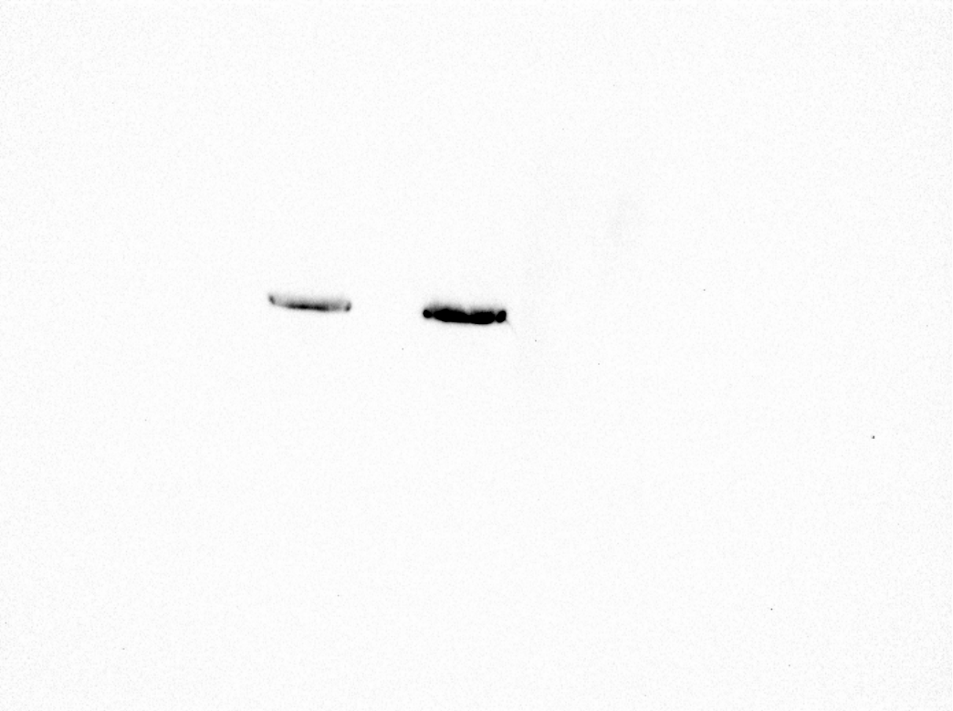


Exposure: 20 sec

**c**


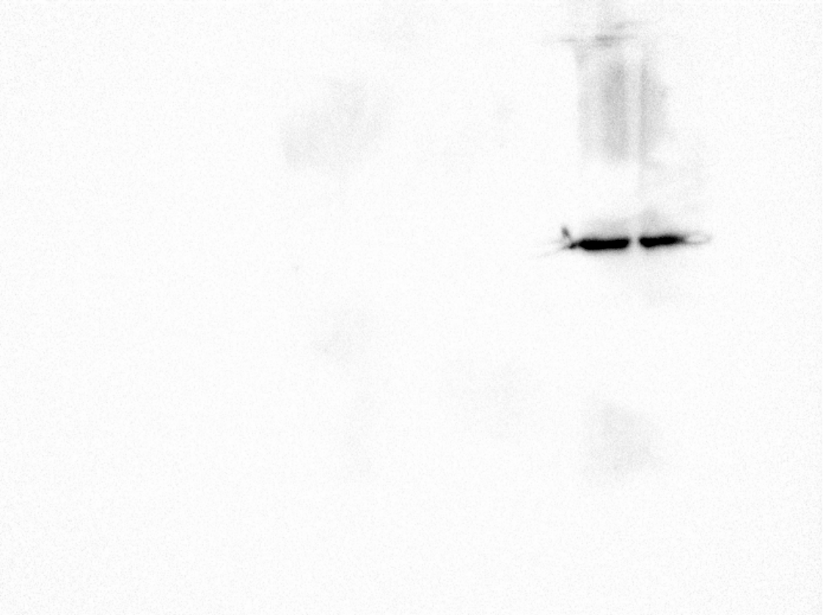


Exposure: 0.5 sec


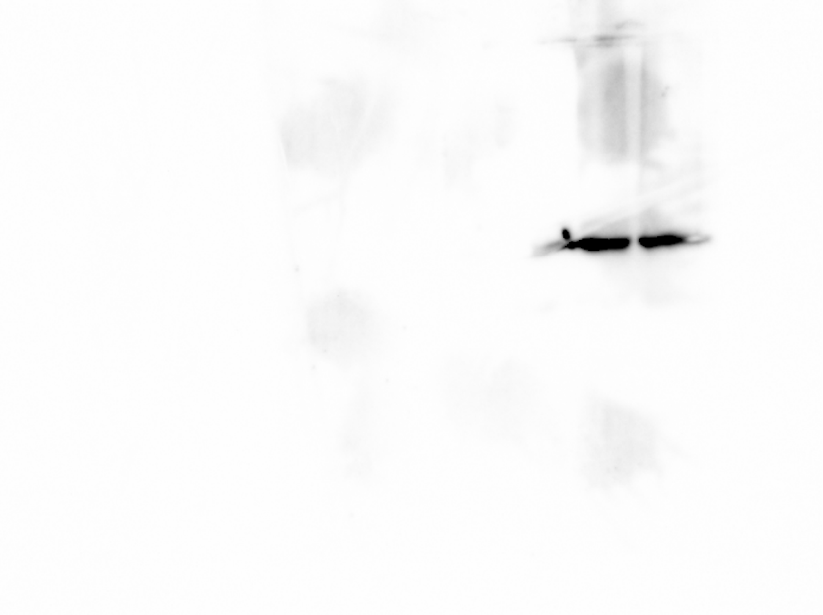


Exposure: 5 sec


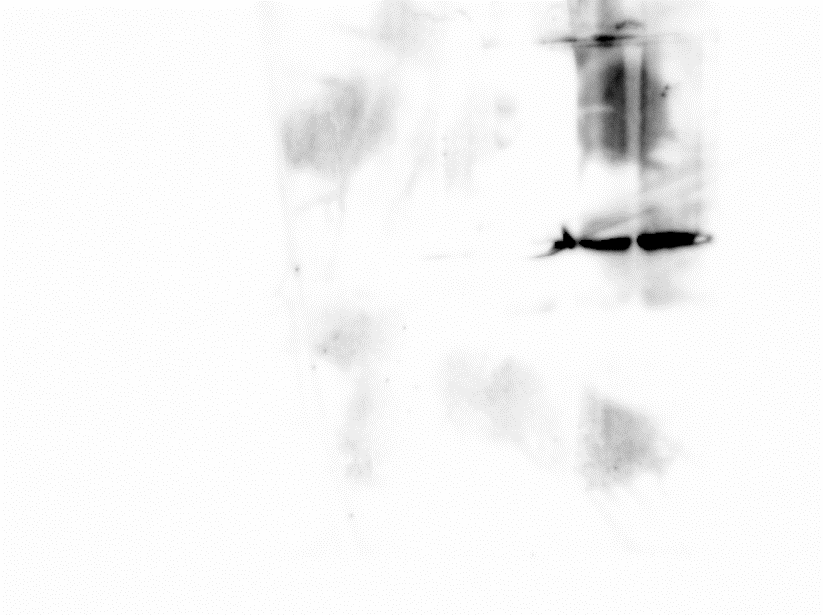


Exposure: 20 sec

**d**


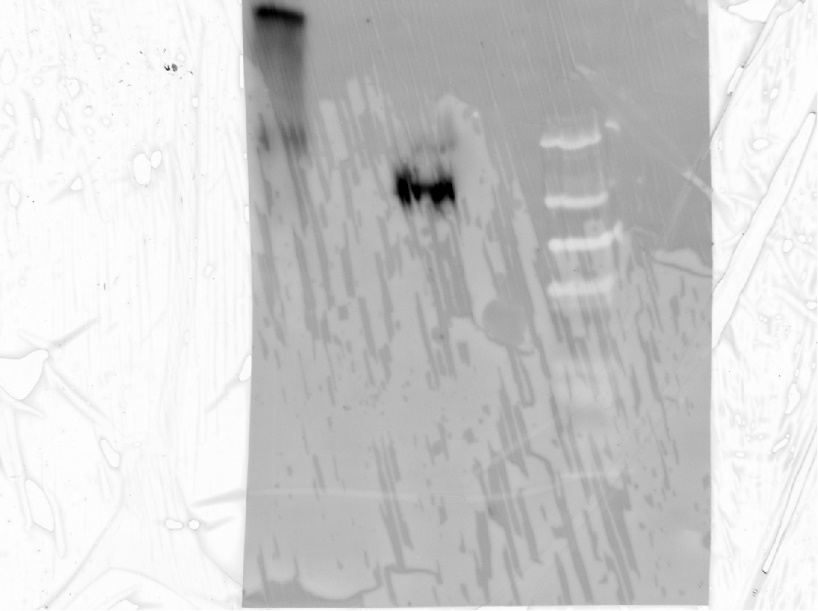


**Supplementary Figure S4. Original, unedited blot images with multiple exposures.** Multiple exposures of blot images shown in Supplementary Figures S2. Membrane blots were probed for a) mouse anti-SARS-CoV-2 S1 spike antibody (Cat# MAB105403, R&D systems), b) mouse anti-VSV-G (Cat #MABF2321, Sigma Aldrich), c) mouse anti-β-actin (GenScript cat #A00702), and d) representative image of protein ladder in one of the blots. Blots were then treated with secondary rabbit anti-mouse IgG-HRP (Cat# 610-4302, Rockland) and developed with enhanced chemiluminescence (ECL) reagents (catalog number NEL105001EA; PerkinElmer). Blot was imaged on Bio-Rad ChemiDoc XRS+ imaging system. Dashed outline indicates membrane edges.
